# *Bordetella hinzii* Pneumonia in Patient with SARS-CoV-2 Infection

**DOI:** 10.3201/eid2804.212564

**Published:** 2022-04

**Authors:** Hend Ben Lakhal, José Bras Cachinho, Pierre Kalfon, Thierry Naas, Zehaira Benseddik

**Affiliations:** Centre Hospitalier de Chartres, Le Coudray, France (H. Ben Lakhal, J. Bras Cachinho, P. Kalfon, Z. Benseddik);; Hôpital Bicêtre, Kremlin-Bicêtre, France (T. Naas)

**Keywords:** Bordetella hinzii, bacteria, pneumonia, infection, SARS-COV-2, severe acute respiratory syndrome coronavirus 2, coronaviruses, virus, coronavirus disease, COVID-19, respiratory infections, pulmonary aspergillosis, zoonoses, France

## Abstract

Patients infected with severe acute respiratory syndrome coronavirus 2 might have bacterial and fungal superinfections develop. We describe a clinical case of coronavirus disease with pulmonary aspergillosis associated with *Bordetella hinzii* pneumonia in an immunocompetent patient in France. *B. hinzii* infections are rare in humans and develop secondary to immunosuppression or debilitating diseases.

Severe acute respiratory syndrome coronavirus 2 (SARS-COV-2) has spread globally and strained health systems with an exponentially increasing number of acute respiratory failures (*1*). Because severe cases of respiratory distress require ventilator assisted respiration, severe bacterial and fungal co-infections can develop and lead to increased deaths (*2*,*3*). *Bordetella hinzii* is a gram-negative, aerobic coccobacillus initially described as a cause of respiratory infection in poultry and rarely in rodents (*4*,*5*). Human infections are rare and occur mostly in immunocompromised persons upon exposure to infected animals (*6*,*7*). In humans, these infections were described in 1994 in an HIV-infected patient as a cause of bacteremia (*6*) and have since been rarely identified in a wide range of infections (*8*–*10*). We report a clinical case of SARS-COV-2 infection associated with pulmonary aspergillosis and *B. hinzii* pneumonia.

## The Study

This case-patient was identified during routine patient care. Thus, the need for ethics approval was exempted; verbal consent was obtained from the patient.

A 63-year-old man with no notable medical history was admitted for cough, asthenia, and shortness of breath starting 3 days before admission. The patient had a positive result for a SARS-CoV-2 rapid antigen autotest. At the emergency department, COVID-19 diagnosis was confirmed by a positive SARS-CoV-2 RT-PCR result for a nasopharyngeal swab sample and chest computed tomography, which showed bilateral ground-glass opacities (50% involvement) and beginning of consolidation in the lower lobes of the lungs (Figure).

**Figure:**
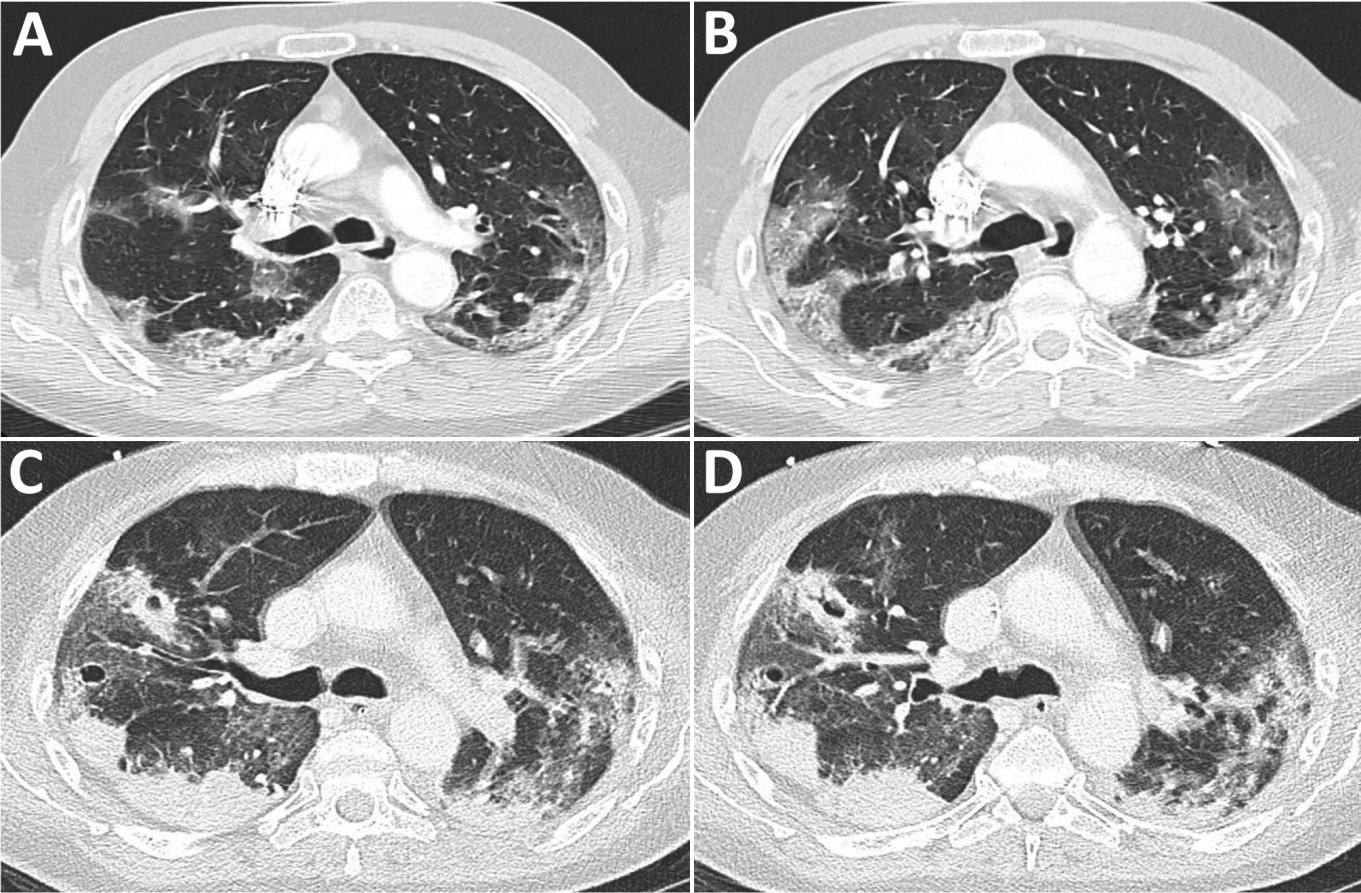
Computed tomography scans of patient with *Bordetella hinzii* pneumonia and severe respiratory syndrome coronavirus 2 infection. A, B) Scan at admission showing bilateral evidence of extensive areas of mainly crazy paving patterns with some posterior consolidations. C, D) Scan at day 25 showing marked increased extent of consolidation.

He received dexamethasone (6 mg/d for 10 d), subcutaneous, low molecular weight heparin (2 × 6,000 IU/d during the entire hospitalization), ceftriaxone (2 g/d), and spiramycin (1.5 × 10^6^ IU 3×/d). On day 2 of hospitalization, he was transferred to the intensive care unit, antimicrobial drug treatment was stopped, and awake prone positioning was combined with high-flow nasal oxygen therapy.

On day 9, mechanical ventilation was applied because of acute respiratory distress syndrome, worsening hypoxemia, and gas exchange deterioration. There was no documented bacterial superinfection, and after 48 hours, the patient’s oxygenation level had improved.

On day 13, respiratory function worsened; purulent aspiration and fever developed, and inflammatory markers increased (C-reactive protein 254 mg/L [reference <10 mg/L] and procalcitonin 0.35 ng/mL [reference <0.1 ng/mL]). Four-day intravenous piperacillin/tazobactam treatment (4 g/0.5 g 4×/d) was initiated, and an endotracheal aspirate (EA) showed oropharyngeal flora (10^7^ CFU/mL) and 5 × 10^5^ CFU/mL methicillin-susceptible *Staphylococcus aureus*, 5 × 10^3^ CFU/mL *B. hinzii*, 5 × 10^2^ CFU/mL amoxicillin-susceptible *Escherichia coli*, and 5 × 10^2^ CFU/mL *Candida tropicalis.*

On day 17, another EA showed oropharyngeal flora (10^7^ CFU/mL), decreased methicillin-susceptible *S. aureus* (5 × 10^3^ CFU/mL), increased *B. hinzii* (10^6^ CFU/mL), amoxicillin-susceptible *E. coli* (10^7^ CFU/mL), and *Aspergillus fumigatus* (10^2^ CFU/mL). *A. fumigatus* was considered as an infection because of worsening of respiratory failure despite piperacillin/tazobactam treatment, ventilatory support for severe acute respiratory distress syndrome, an *A.* *fumigatus*‒positive culture on EA (absent on previous EAs), and a computed tomography scan showing cavitating infiltrates (Figure). Voriconazole treatment (400 mg on the first day, followed by 200 mg/12 h) was given for 21 days, in combination with intravenous co-amoxiclav (1 g 3×/d).

EA was repeated on day 25 because of persistence of fever, progressive clinical deterioration, and worsening of radiologic findings and showed 10^6^ CFU/mL *B. hinzii*, 10^5^ CFU/mL amoxicillin-susceptible *E. coli*, and 10^3^ CFU/mL *C. tropicalis,* which was considered as colonization. We switched treatment to piperacillin/tazobactam (4 g/0.5 g 4×/d for 8 d), which resulted in negative results on subsequent EA samples. Testing of rectal swab samples, blood, and urine cultures remained negative throughout hospitalization. The patient was extubated on day 46 and discharged uneventfully from the hospital.

*B. hinzii* grew on horse blood agar (bioMérieux, https://www.biomerieux.com) at 37°C after incubation for 24 hours as smooth, gray colonies. We identified *B. hinzii* by using matrix-assisted laser desorption/ionization time-of-flight mass spectrometry (Biotyper; Bruker, https://www.bruker.com) and confirmed it by using whole-genome sequencing (Illumina, https://www.illumina.com) as described (*11*). 

We initially performed antimicrobial susceptibility testing by using Etest (bioMérieux), confirmed results by using disk diffusion and broth microdilution (Thermo Fisher Sensititer System; Thermo Fisher, https://www.thermofisher.com) (Table), and interpreted results by using the 2021 European Committee on Antimicrobial Susceptibility Testing pharmacokinetic/pharmacodynamic (nonspecies related) breakpoints (*12*). *B. hinzii* CHAR-1 showed resistance to amoxicillin, cefotaxime, aminoglycosides, and ciprofloxacin; intermediate resistance to amoxicillin/clavulanic acid and ceftazidime; and susceptibility to piperacillin/tazobactam, meropenem, and imipenem. New molecules were also tested and remained susceptible, except for ceftolozane/tazobactam (Table).

**Table:** Antimicrobial susceptibility of *Bordetella hinzii* isolate from patient with pneumonia and severe respiratory syndrome coronavirus 2 infection, by Etest and broth microdilution*

Test and antimicrobial drug	MIC for Etest, μg/mL	MIC for BMD, μg/mL	Interpretation	EUCAST PK-PD breakpoint, mg/L†	
S	R
Routine antibiogram					
Amoxicillin	32	ND	R	<2	>8
Amoxicillin/clavulanic acid (2)‡	6	ND	I	<2	>8
Ticarcillin/clavulanic acid	16	ND	I	<8	>16
Piperacillin/tazobactam (4)‡	0.5§	<4	S	<8	>16
Cefotaxim	>32	ND	R	<1	>2
Ceftazidime	2	ND	S	<4	>8
Aztreonam	>256	>32	R	<4	>8
Cefepime	4	4	S	<4	>8
Meropenem	0.06	0.25	S	<2	>8
Imipenem	0.75	<1	S	<2	>4
Ciprofloxacin	0.75	ND	R	<0.25	>0.5
Levofloxacin	0.75	ND	I	<0.5	>1
Amikacin	8	2	R	<1	>1
Gentamicin	2	ND	R	<0.5	>0.5
Tobramycin	8	>4	R	<0.5	>0.5
Additional antimicrobial drugs tested					
Imipenem/relebactam (4)‡	ND	1	S	<2	>2
Meropenem/vaborbactam (8)‡	ND	0.12	S	<8	>8
Ceftazidime/avibactam (4)‡	ND	4	S	<8	>8
Cefiderocol	ND	1	S	<2	>2
Ceftolozane/tazobactam (4)‡	ND	8	R	<4	>4
Tigecycline	ND	0.5	S	0.5	>0.5
Eravacycline	ND	0.12	IE	IE	IE
Fosfomycin	ND	>64	IE	IE	IE
Colistin	ND	<0.5	IE	IE	IE

*BMD, broth microdilution; EUCAST, European Committee on Antimicrobial Susceptibility Testing; I, intermediate/susceptible with high dose; IC, inhibitory concentration; IE, insufficient evidence; ND, not determined; PK/PD, pharmacokinetic/pharmacodynamic; R, resistant; S, susceptible. †MIC breakpoints were interpreted by using EUCAST guidelines and PK-PD (nonspecies related) breakpoints (https://www.eucast.org/clinical_breakpoints). ‡Numbers in parentheses indicate the amount of inhibitor used: 2 μg/mL for clavulanic acid; 4 μg/mL for tazobactam, relebactam, and avibactam; and 8 μg/mL for vaborbactam. §Piperacillin/tazobactam MIC was determined by using the PIP/Tazo UMIC strip (Biocentric, https://biocentricinc.com).

We identified β-lactamase activity by using *B. hinzii* CHAR-1 crude protein extracts as described (*13*). In silico analysis showed a β-lactamase, Hinzii *Bordetella* lactamase (HBL-1), which had all canonical boxes of a functional broad-spectrum class A penicillinase (*13*) (Appendix Figure). HBL-1 had 62.7% amino acid identity with BOR-1 β-lactamase from *B. parapertussis* (*13*). Highly related sequences (99.7%‒100% amino acid identity) were identified in genome sequences of *B. hinzii* available in public databases, suggesting that HBL-1–like enzymes might be native to that species (Appendix Figure). MICs of aminopenicillins and carboxypenicillins might be explained by expression of HBL-1, but, as suggested for *B. parapertussis*, additional nonenzymatic β-lactam resistance mechanisms, such as impermeability, efﬂux, or penicillin-binding protein afﬁnities, might be associated for *B. hinzii*. The complete genome and HBL-1 sequences have been deposited in DDBJ/EMBL (accession no. JAJTJL000000000) and GenBank (accession nos. OM212391).

The lung microbiota of deceased patients who had COVID-19 showed complex bacterial and fungal colonization by opportunistic pathogens (*14*). SARS‐CoV‐2 infection, antimicrobial drug pressure, alveolar damage, persistent lymphocytic depletion, mechanical ventilation, corticosteroid therapy, and prolonged hospital stays might predispose critically ill COVID-19 patients to opportunistic bacterial or fungal superinfection (*2*,*14*). Critically ill COVID-19 patients have the highest percentage of secondary pulmonary infections (34.5%) compared with percentages for severely ill (8.3%) and moderately ill (3.9%) COVID-19 patients (*15*). COVID-19‒associated pulmonary aspergillosis is a worrisome complication in critically ill patients who show increased illnesses and deaths (*2*). *A. fumigatus* co-infections are frequent among critically ill COVID-19 patients (*2*,*3*). Rothe et al. showed that in a group of 50 critically ill COVID-19 patients admitted to the intensive care unit, 34% were co-infected with Enterobacterales and 18% with *A. fumigatus* (*15*).

Human cases of *B. hinzii* infection are rare and associated mostly with immunosuppression and anterior poultry exposure (*7*–*10*). Our patient reported recent exposure to geese and ducks, which probably led to latent or chronic colonization (digestive or respiratory tract) before infection at a time when he was most vulnerable (e.g., COVID-19 and aspergillosis superinfection). Reports of patients who have *B. hinzii* infections seem to be increasing in recent years, which might reflect emergence of this pathogen or availability of better identification methods, such as matrix-assisted laser desorption/ionization time-of-flight mass spectrometry, 16S rRNA gene sequencing, and whole-genome sequencing (*8*). Among the few *B. hinzii* infections described, none reported *Aspergillus* infections (*9*).

Reported *B. hinzii* isolates were frequently multidrug resistant, including resistance to cephalosporins, aminoglycosides, and quinolones, but remained susceptible to piperacillin/tazobactam, ceftazidime, tigecycline, and meropenem (*9*,*10*). Interpretation of antimicrobial susceptibility testing is not established, and the choice of antimicrobial drugs and treatment duration are not standardized. Cases with documented pneumonia were successfully treated with piperacillin/tazobactam or cefmetazole (*9*). Our patient was successfully treated with piperacillin/tazobactam, but treatment with amoxicillin/clavulanic acid failed, probably because of intermediate susceptibility of *B. hinzii* to this antimicrobial drug. Our study suggests that *B. hinzii* needs to be taken into account when initiating antimicrobial drug therapy.

## Conclusions

Increasing reports of invasive *B. hinzii* might indicate its emergence as a pathogen in immunocompromised patients. We describe a *B. hinzii* and *A. fumigatus* co-infection in a SARS-CoV-2‒infected immunocompetent patient who had no underlying conditions but had probable transient immunosuppression caused by dexamethasone treatment and SARS-CoV-2 infection. Our study highlights the role of opportunistic infections (by fungal or rare bacterial species) in COVID-19 patients and the need to serially monitor the bacteria/fungi in the lower respiratory tract for timely personalized treatment.

Appendix Additional information on *Bordetella hinzii* pneumonia in patient with SARS-CoV-2 infection.
