# Supplementary material for: Bordetella hinzii Pneumonia in Patient with SARS-CoV-2 Infection
Source: Emerg Infect Dis. 2022 Apr;28(4):844–7. doi: 10.3201/eid2804.212564 (PMC8962905; doi:10.3201/eid2804.212564)
Supplement: Appendix — Additional information on Bordetella hinzii pneumonia in patient with SARS-CoV-2 infection. [file 21-2564-Techapp-s1.pdf]

Article DOI: https://doi.org/10.3201/eid2804.212564

Bordetella hinzii Pneumonia in Patient with SARS-CoV-2 Infection

Appendix

```
BOR-1 MNRRGFGAGMLAALGAACMPPWARAGVRRAARPADAAAQAQRQLALLEQRHGARLGVQVQ   60
PBL-1 MDRRTFGAGVLAWLGASAAGLPALAGVRDSLS--AAGDDAQRQLARLEAREGGRLGVSLL   58
HBL-1 MDRRTFGAGVLAWLGASAAGLPALAGVRDSLP--AASDDAQRQLARLEAREGGRLGVSLL   58
HBL-2 MDRRTFGAGVLAWLGASAAGLPALAGVRDSLP--AASDDAQRQLARLEAREGGRLGVSLL   58
HBL-3 MDRRTFGAGVLAWLGASAAGLPALAGVRDSLP--AASDDAQRQLARLEAREGGRLGVSLL   58
      *:** ****:** ***:.    * **** :     *. :****** ** *.*.****.:

BOR-1 DRDSGGAFSHRADERFPLCSTFKLLAAAAVLARADRGDDSLARLIRYGATDIVAYSPVTG   120
PBL-1 DVQSGYAIAYRADERFALCSTFKLLAVGAVLTRVARGEDDLSRPMRLSAADIVTYSPVTQ   118
HBL-1 DVQSGYAIAYRADERFALCSTFKLLAVGAVLTRVARGEDDLSRPMRLSAADIVDYSPVTQ   118
HBL-2 DVQSGYAIAYRADERFALCSTFKLLAVGAVLTRVARGEDDLSRPMRLSAADIVDYSPVTQ   118
HBL-3 DVQSGYAIAYRADERFALCSTFKLLAVGAVLTRVARGEDDLSRPMRLSAADIVDYSPVTQ   118
      * :** *:::****** *********..***:*. **:*.*:* :* .*:*** *****
                       S70 K73

BOR-1 PRQAEGMTLEQLCEAAVTRSDNTAGNLLLSTLGGPPGLTAYARGLGDRVTRLDRIETALN   180
PBL-1 QRLNEGMTLGQLCEAALLWGDNTAANLLLSTIGGPPGLTAYARALGDGVTRLDRLETALN   178
HBL-1 QRLNEGMTLGQLCEAALLWGDNTAANLLLSTIGGPPGVTAYARALGDGATRLDRLETALN   178
HBL-2 QRLNEGMTLGQLCEAALLWGDNTAANLLLSTIGGPPGVTAYARALGDGATRLDRLETALN   178
HBL-3 QRLNEGMTLGQLCEAALLWGDNTAANLLLSTIGGPPGVTAYARALGDGATRLDRLETALN   178
       *  ***** ******:  .****.******:*****:*****.*** .*****:*****
                       S130  N136                      R164 E166

BOR-1 EARPGDPRDTTTPAAMAGNLERLLLGDALQPASRQRLADWLLASRTGDTRLRAGLPSGWR   240
PBL-1 EARPGDERDTTTPAAMLGNLRQLVLGDVLPAPERERLRDWLMQCRTGRERLRAGLPAAWA   238
HBL-1 EARPGDERDTTTPAAMLGNLRQLVLGDALPAPERERLRDWLMQCRTGQQRLRAGLPAGWS   238
HBL-2 EARPGDERDTTTPAAMLGNLRQLVLGDALPAPERERLRDWLMQCRTGQQRLRAGLPASWS   238
HBL-3 EARPGDERDTTTPAAMLGNLRQLVLGDALPAPERERLRDWLMQCRTGQQRLRAGLPAGWS   238
      ****** ********* ***.:*:***.*   .*:** ***: .***  *******:.*
          D179

BOR-1 IGDKTGAGGNGTNNDVGVIWPRDGAPVLITAYLTQSSASRETQNAVLAEVGRIAAHAVAA   300
PBL-1 LAHRSGAGGHGSCNDIGVAWPAPAAPVLISAYLTESPLDLPGRERVLAEAARILAHALVS   298
HBL-1 LGHRTGAGGHGTCNDIGVAWPTPTTPVVISVYLTESPLDLPGRERVLAEAARILAHALAS   298
HBL-2 LGHRTGAGGHGTCNDIGVAWPTPTTPVVISVYLTESPLDLPGRERVLAEAARILAHALAS   298
HBL-3 LGHRTGAGGHGTCNDIGVAWPTPTTPVVISVYLTESPLDLPERERVLAEAARILAHALAS   298
      :..::****:*: **:** **   :**:*:.***:*  .   :: ****..** ***:.:
       R/K234

BOR-1 WRLGG-   305
PBL-1 ARLHAG   304
HBL-1 ARLHAG   304
HBL-2 ARLHAG   304
HBL-3 ARLHAG   304
       ** .
```

Appendix Figure. Sequence alignment of β-lactamases BOR-1 from *Bordetella parapertussis* (WP_033463656 [*1*]); PBL-1 (*Pseudohinzii Bordetella* Lactamase) from *B. pseudohinzii* (WP_068945286), HBL-1 from *B. hinzii* CHAR-1 (this study, accession number pending and WP_080700357.1), HBL-2 from *B. hinzii* (WP_142176586.1), and HBL-3 from *B. hinzii* (WP_029580329.1). Conserved boxes in class A β-lactamases are indicated by black boxes, residues involved in hydrolysis or substrate stabilization in the active site are highlighted in gray, and the Omega loop is underlined (*2*). Amino acid differences among *B. hinzii* β-lactamases are indicated in bold. S130G substitution has been shown to be involved in inhibitor resistance (*3*).

References

1. Lartigue MF, Poirel L, Fortineau N, Nordmann P. Chromosome-borne class A BOR-1 beta-Lactamase of *Bordetella bronchiseptica* and *Bordetella parapertussis.* Antimicrob Agents Chemother. 2005;49:2565–7. PubMed https://doi.org/10.1128/AAC.49.6.2565-2567.2005

2. Verma D, Jacobs DJ, Livesay DR. Variations within class-A β-lactamase physiochemical properties reflect evolutionary and environmental patterns, but not antibiotic specificity. PLOS Comput Biol. 2013;9:e1003155. PubMed https://doi.org/10.1371/journal.pcbi.1003155

3. Cantón R, Morosini MI, de la Maza OM, de la Pedrosa EG, De la Pedrosa EG. IRT and CMT beta-lactamases and inhibitor resistance. Clin Microbiol Infect. 2008;14(Suppl 1):53–62. PubMed https://doi.org/10.1111/j.1469-0691.2007.01849.x
